# Which Approach Is Better for Minimally Invasive Surgery

**DOI:** 10.31662/jmaj.2020-0101

**Published:** 2021-01-14

**Authors:** Masafumi Inomata

**Affiliations:** 1 Department of Gastroenterological and Pediatric Surgery, Oita University Faculty of Medicine, Yufu, Japan

**Keywords:** minimally invasive surgery, colorectal cancer, robotic surgery

I recently read with interest the review of minimally invasive surgery for colorectal cancer by Yamauchi S et al. ^(1)^. This is a reasonable review to evaluate the efficacy of laparoscopic, robotic, and TaTME surgery for colon and rectal cancer in the JMA Journal. For new surgical procedures to become widely adopted, first, technical safety has to be confirmed. Thereafter, these procedures have to be shown to be superior in long-term outcomes compared with established procedure.

In terms of laparoscopic sugary for colon cancer, the technical and oncological safety seems to be considered acceptable worldwide. To date, there is still no conclusive data about which approach is better between open surgery and laparoscopic surgery. Several reports supported the feasibility of long-term outcomes in obese patients with colon cancer in laparoscopic cancer resection compared with open resection. In contrast, several studies reported that laparoscopic surgery for colon cancer is technically more demanding in obese patients than non-obese patients, and special care was required because of the increased risk of developing postoperative complications. From the subgroup analysis of JCOG0404, in the high BMI subgroup (BMI ≥25.0 kg/m^2^), the adjusted HR (95% CI) of clinical factors for LAP (n = 133) versus OP (n = 121) was 3.37 (1.24-9.19) for OS and 2.95 (1.53-5.69) for RFS. The long-term outcome of LAP compared with OP for colon cancer with curative resection was significantly poorer ^(2)^. However, these findings might not provide concrete evidence of the superiority of OP over LAP for obese colon cancer patients, for which there are several reasons. First, the numbers of patients with recurrence in the OP and LAP groups were 12 and 30 patients, respectively, which were insufficient to obtain concrete evidence. Second, the present subgroup analysis of the first site of recurrence showed that no increase in the incidence of peritoneum, lymph node metastatic recurrence, or local recurrence was noted in the LAP group compared with the OP group. Third, because of the insufficient number of patients with a higher BMI, evaluation of the correlation between higher BMI and worse long-term outcomes could not be performed. Thus, a multicenter, retrospective, large-scale study initiated by more than 50 member institutions of the Japan Society of Laparoscopic Colorectal Surgery is currently being conducted to compare the short- and long-term outcomes of colorectal cancer patients with high BMI (≥25.0 kg/m^2^) undergoing LAP or OP (trial number: UMIN000033529) ^(3)^.

When considering technical and oncological safety of laparoscopic surgery for rectal cancer, circumferential resection margin (CRM) status is one of the most important parameter to assess. However, Japanese Classification of Colorectal, Appendiceal, and Anal Carcinoma have no description about CRM. Presently, RM (radial margin) is widely used as a daily practice in Japan. It is said that evaluating CRM needs more effort and time for pathologists. To create evidence about surgery for rectal cancer in Japan, a multicenter, prospective, large-scale study initiated by more than 20 member institutions is currently being conducted to evaluate the CRM parameter (PRODUCT trial number: UMIN 000034364) ^(4)^. I consider that evaluating CRM in open, laparoscopic, TaTMA, and robotic surgery for rectal cancer is a key for evaluating the clinical outcome, which can be assessed as a short-term outcome as well as be associated with long-term outcome.

With regard to robotic surgery for rectal cancer, it is still a problem that the apparent superiority to laparoscopic surgery is unclear. Presently, robotic surgery remains an investigational technique that should only be performed by experienced surgeons in high-volume centers.

Thirty years has passed since the emerging laparoscopic surgery as minimally invasive surgery (MIS). Nowadays, we have several approaches for colorectal cancer for MIS. The indication for each approach should be determined at each surgical team level. However, for example, determining the indication for laparoscopic surgery in each hospital based on each surgical team level is nonobjective and difficult. Therefore, Endoscopic Surgical Skill Qualification (ESSQ) system by Japan Society for Endoscopic Surgery could help surgeons determine the indications for laparoscopic surgery in each hospital. An operation performed by ESSQS-certified surgeons is one of the most valuable predictors of optimal clinical outcome in terms of anastomotic leakage after laparoscopic surgery for rectal cancer ^(5)^. Moreover, to introduce, maintain, and improve the quality of minimally invasive surgery, training course should be conducted, which might become essential to enhance the education system to spread MIS properly. Gradually, further clinical outcomes about MIS will be reported to clarify the positioning of laparoscopic and robotic surgery in the future.

In conclusion, the present report showed us that the accumulation of higher quality evidence can help in deciding whether to perform minimally invasive surgery, including robotic surgery and transanal total mesorectal excision for rectal cancer.

## Article Information

### Conflicts of Interest

None

### Disclaimer

Masafumi Inomata is one of the Editors of JMA Journal and on the journal's Editorial Staff. He was not involved in the editorial evaluation or decision to accept this article for publication at all.
